# Supplementary material for: Fitness level, but not sex, affects exercise-induced pain modulation
Source: eLife. 2026 Oct 30;15:RP109061. doi: 10.7554/eLife.109061 (PMC13722413; doi:10.7554/eLife.109061)
Supplement: Supplementary file 2 [file elife-109061-supp1.docx]

**Table S1**. Full LMER model output of exercise intensity and stimulus intensity on behavioural overall pain ratings.

| Fixed Effects | Estimate | *SE* | *df* | *t* | *P* |
| --- | --- | --- | --- | --- | --- |
| Intercept | 74.43 | 4.96 | 58.99 | 15.00 | **2×10^-16^** |
| Exercise Intensity | -3.29 | 2.14 | 1133.64 | -1.53 | 0.13 |
| Trial | -0.58 | 0.20 | 1121.78 | -2.94 | **0.003** |
| Block | 1.49 | 1.14 | 1130.36 | 1.30 | 0.19 |
| LMER = linear mixed effects model, *SE* = standard error, *df* = degrees of freedom. Subject was included as random effect. Trial and block were included as fixed effects. *N* = 21. | | | | | |

**Table S2**. Full LMER model output of exercise intensity and stimulus intensity on behavioural pressure pain ratings.

| Fixed Effects | Estimate | *SE* | *df* | *t* | *P* |
| --- | --- | --- | --- | --- | --- |
| Intercept | 85.25 | 6.36 | 37.20 | 13.40 | **8.19 ×10^-13^** |
| Exercise Intensity | -3.05 | 2.15 | 570.12 | -1.42 | 0.16 |
| Trial | -0.63 | 0.20 | 567.02 | -3.15 | **0.002** |
| Block | 1.59 | 1.14 | 569.48 | 1.39 | 0.16 |
| LMER = linear mixed effects model, *SE* = standard error, *df* = degrees of freedom. Subject was included as random effect. Trial and block were included as fixed effects. *N* = 21. | | | | | |

**Table S3**. Post-hoc *t*-tests comparing exercise intensities (LI and HI) for pressure pain (across all stimulus intensities).

| Fixed Effects | T | *SE* | *df* | *t-ratio* | *P* |
| --- | --- | --- | --- | --- | --- |
| LI vs. HI | 3.05 | 2.15 | 570 | 1.42 | 0.16 |
| *SE* = standard error, *df* = degrees of freedom. LI = low-intensity, HI = high-intensity. | | | | | |

**Table S4**. Full LMER model output of exercise intensity and stimulus intensity on behavioural heat pain ratings.

| Fixed Effects | Estimate | *SE* | *df* | *t* | *P* |
| --- | --- | --- | --- | --- | --- |
| Intercept | 64.23 | 6.67 | 54.04 | 9.64 | **2.47×10^-11^** |
| Exercise Intensity | -4.13 | 2.93 | 538.70 | -1.41 | 0.16 |
| Trial | -0.47 | 0.27 | 531.96 | -1.76 | 0.08 |
| Block | 1.01 | 1.56 | 535.90 | 0.65 | 0.52 |
| LMER = linear mixed effects model, *SE* = standard error, *df* = degrees of freedom. Subject was included as random effect. Trial and block were included as fixed effects. *N* = 20. | | | | | |

**Table S5**. Post-hoc *t*-tests comparing exercise intensities (LI and HI) for heat pain (across all stimulus intensities).

| Fixed Effects | T | *SE* | *df* | *t-ratio* | *P* |
| --- | --- | --- | --- | --- | --- |
| LI vs. HI | ´4.13 | 2.93 | 539 | 1.41 | 0.16 |
| *SE* = standard error, *df* = degrees of freedom. LI = low-intensity, HI = high-intensity. | | | | | |

**Table S6**. Full LMER model output of exercise intensity and stimulus intensity on behavioural overall pain ratings.

| Fixed Effects | Estimate | *SE* | *df* | *t* | *P* |
| --- | --- | --- | --- | --- | --- |
| Intercept | 4.70 | 5.84 | 100.88 | 0.80 | 0.42 |
| Exercise Intensity | 7.76 | 5.23 | 1120.67 | 1.48 | 0.14 |
| Stimulus Intensity | 1.45 | 0.07 | 1120.31 | 19.62 | **<2×10^-16^** |
| Trial | -0.79 | 0.16 | 1119.86 | -5.08 | **4.45×10^-7^** |
| Block | 1.55 | 0.90 | 1125.10 | 1.74 | 0.08 |
| Stimulus Intensity x Exercise Intensity | -0.22 | 0.10 | 1119.88 | -2.22 | **0.03** |
| LMER = linear mixed effects model, *SE* = standard error, *df* = degrees of freedom. Subject was included as random effect. Trial and block were included as fixed effects. *N* = 21. | | | | | |

**Table S7**. Full LMER model output of exercise intensity and stimulus intensity on behavioural pressure pain ratings.

| Fixed Effects | Estimate | *SE* | *df* | *t* | *P* |
| --- | --- | --- | --- | --- | --- |
| Intercept | 31.70 | 6.61 | 48.23 | 4.79 | **1.61×10^-5^** |
| Exercise Intensity | 1.27 | 4.72 | 565.15 | 0.27 | 0.79 |
| Stimulus Intensity | 1.13 | 0.07 | 565.20 | 17.04 | **<2×10^-16^** |
| Trial | -0.91 | 0.14 | 565.02 | -6.44 | **2.61×10^-10^** |
| Block | 1.60 | 0.81 | 566.32 | 2.00 | **0.05** |
| Stimulus Intensity x Exercise Intensity | -0.09 | 0.09 | 565 | -0.97 | 0.33 |
| LMER = linear mixed effects model, *SE* = standard error, *df* = degrees of freedom. Subject was included as random effect. Trial and block were included as fixed effects. *N* = 21. | | | | | |

**Table S8**. Post-hoc *t*-tests (Tukey-adj.) for LMER models in pressure pain comparing the averaged pain ratings at HI and LI exercise at each stimulus intensity.

|  | *T* | *SE* | *df* | *T-ratio* | *P* |
| --- | --- | --- | --- | --- | --- |
| VAS 30 | 1.73 | 2.54 | 564 | 0.68 | 0.50 |
| VAS 50 | 2.38 | 2.54 | 564 | 0.94 | 0.35 |
| VAS 70 | 5.27 | 2.63 | 564 | 2.01 | **0.05** |
| SE = standard error, df = degrees of freedom. LI = low-intensity, HI = high-intensity. | | | | | |

**Table S9.** Full LMER model output of exercise intensity and stimulus intensity on behavioural heat pain ratings.

| Fixed Effects | Estimate | *SE* | *df* | *t* | *P* |
| --- | --- | --- | --- | --- | --- |
| Intercept | -21.39 | 7.23 | 58.68 | -2.96 | **0.004** |
| Exercise Intensity | 14.03 | 5.71 | 530.24 | 2.46 | **0.01** |
| Stimulus Intensity | 1.76 | 0.08 | 530.58 | 21.64 | **<2×10^-16^** |
| Trial | -0.54 | 0.17 | 529.92 | -3.19 | **0.001** |
| Block | 1.08 | 0.99 | 531.34 | 1.10 | 0.27 |
| Stimulus Intensity x Exercise Intensity | -0.36 | 0.11 | 529.95 | -3.32 | **0.001** |
| LMER = linear mixed effects model, *SE* = standard error, *df* = degrees of freedom. Subject was included as random effect. Trial and block were included as fixed effects. *N* = 20. | | | | | |

**Table S10**. Post-hoc *t*-tests (Tukey-adj.) for LMER models in heat pain comparing the pain ratings at HI and LI exercise at each stimulus intensity.

|  | *T* | *SE* | *df* | *T-ratio* | *P* |
| --- | --- | --- | --- | --- | --- |
| VAS 30 | -2.78 | 3.00 | 529 | -0.93 | 0.35 |
| VAS 50 | 3.37 | 3.00 | 529 | 1.13 | 0.26 |
| VAS 70 | 12.02 | 3.19 | 529 | 3.77 | **0.0002** |
| SE = standard error, df = degrees of freedom. LI = low-intensity, HI = high-intensity. | | | | | |

**Table S11.** Full LMER model output of exercise intensity and subgroup on behavioural heat pain ratings (averaged across all stimulus intensities).

| Fixed Effects | | Estimate | *SE* | *df* | *t* | *P* |
| --- | --- | --- | --- | --- | --- | --- |
| Intercept | 55.96 | | 5.94 | 115.57 | 9.42 | **5.59×10^-16^** |
| Exercise intensity | 3.01 | | 3.52 | 1886.00 | 0.86 | 0.392 |
| Group | -11.11 | | 2.70 | 69.06 | -4.11 | **0.0001** |
| Treatment order | 5.57 | | 4.21 | 54.00 | 1.32 | 0.191 |
| Exercise intensity × Group | -1.63 | | 1.68 | 1897.74 | -0.97 | 0.332 |
| LMER = linear mixed effects model, *SE* = standard error, *df* = degrees of freedom. Subject was included as random effect. Trial, block, and treatment order were included as fixed effects. *N* = 59. Subgroup was defined as factor with levels males from previous study, females from previous study, and females from current study. | | | | | | |

**Table S12**. Post-hoc *t*-tests (Tukey-adj.) for LMER models in heat pain (averaged across all stimulus intensities) comparing the pain ratings at HI and LI exercise for each subgroup.

|  | *T* | *SE* | *df* | *T-ratio* | *P* |
| --- | --- | --- | --- | --- | --- |
| Males (previous study) | -0.34 | 2.23 | 1875 | -0.15 | 0.88 |
| Females (previous study) | -1.62 | 2.05 | 1875 | -0.79 | 0.43 |
| Females (current study) | 3.31 | 2.49 | 1918 | 1.33 | 0.18 |
| SE = standard error, df = degrees of freedom. LI = low-intensity, HI = high-intensity. Males (previous study): *n* = 18; females (previous study): *n* = 21; females (current study): *n* = 20. | | | | | |

**Table S13.** Full LMER model output of exercise intensity and subgroup on behavioural heat pain ratings (VAS 70).

| Fixed Effects | Estimate | *SE* | *df* | *t* | *P* |
| --- | --- | --- | --- | --- | --- |
| Intercept | 83.61 | 8.41 | 70.56 | 9.94 | **4.77×10^-15^** |
| Exercise intensity | 11.12 | 3.16 | 560.87 | 3.52 | **0.0005** |
| Subgroup | -14.76 | 4.17 | 57.84 | -3.54 | **0.0008** |
| Treatment order | 10.02 | 6.66 | 53.27 | 1.50 | 0.14 |
| Exercise intensity × Group | -5.96 | 1.53 | 562.24 | -3.89 | **0.0001** |
| LMER = linear mixed effects model, *SE* = standard error, *df* = degrees of freedom. Subject was included as random effect. Trial, block, and treatment order were included as fixed effects. *N* = 59. Subgroup was defined as factor with levels males from previous study, females from previous study, and females from current study. | | | | | |

**Table S14**. Post-hoc *t*-tests (Tukey-adj.) for LMER models in heat pain (VAS 70) comparing the pain ratings at HI and LI exercise for each subgroup.

|  | *T* | *SE* | *df* | *T-ratio* | *P* |
| --- | --- | --- | --- | --- | --- |
| Males (previous study) | -4.35 | 1.97 | 559 | -2.20 | **0.03** |
| Females (previous study) | -0.69 | 1.81 | 559 | 2.90 | 0.71 |
| Females (current study) | 7.95 | 2.32 | 564 | 3.42 | **0.0007** |
| SE = standard error, df = degrees of freedom. LI = low-intensity, HI = high-intensity. Males (previous study): *n* = 18; females (previous study): *n* = 21; females (current study): *n* = 20. | | | | | |

**Table S15.** Full LMER model output of exercise intensity and subgroup on behavioural pressure pain ratings (averaged across all stimulus intensities).

| Fixed Effects | Estimate | *SE* | *df* | *t* | *P* |
| --- | --- | --- | --- | --- | --- |
| Intercept | 46.91 | 6.22 | 83.34 | 7.55 | **5.06×10^-11^** |
| Exercise intensity | 1.54 | 2.79 | 1915.36 | 0.55 | 0.58 |
| Group | -5.34 | 2.99 | 63.00 | -1.79 | 0.08 |
| Treatment order | 3.74 | 4.84 | 55.78 | 0.77 | 0.44 |
| Exercise intensity × Group | -0.86 | 1.32 | 1921.03 | -0.65 | 0.52 |
| LMER = linear mixed effects model, *SE* = standard error, *df* = degrees of freedom. Subject was included as random effect. Trial, block, and treatment order were included as fixed effects. *N* = 60. Subgroup was defined as factor with levels males from previous study, females from previous study, and females from current study. | | | | | |

**Table S16**. Post-hoc *t*-tests (Tukey-adj.) for LMER models in pressure pain (averaged across all stimulus intensities) comparing the pain ratings at HI and LI exercise for each subgroup.

|  | *T* | *SE* | *df* | *T-ratio* | *P* |
| --- | --- | --- | --- | --- | --- |
| Males (previous study) | -0.10 | 1.77 | 1910 | -0.06 | 0.96 |
| Females (previous study) | -0.87 | 1.63 | 1910 | -0.53 | 0.59 |
| Females (current study) | 1.78 | 1.93 | 1929 | 0.92 | 0.36 |
| SE = standard error, df = degrees of freedom. LI = low-intensity, HI = high-intensity. Males (previous study): *n* = 18; females (previous study): *n* = 21; females (current study): *n* = 21. | | | | | |

**Table S17.** Full LMER model output of exercise intensity and subgroup on behavioural pressure pain ratings (VAS 70).

| Fixed Effects | Estimate | *SE* | *df* | *t* | *P* |
| --- | --- | --- | --- | --- | --- |
| Intercept | 68.21 | 6.73 | 91.50 | 10.14 | **<2×10^-16^** |
| Exercise intensity | 2.56 | 3.34 | 576.85 | 0.77 | 0.44 |
| Group | -5.77 | 3.18 | 64.33 | -1.82 | 0.07 |
| Treatment order | 5.31 | 5.07 | 54.94 | 1.05 | 0.30 |
| Exercise intensity × Group | -1.22 | 1.59 | 579.24 | -0.77 | 0.44 |
| LMER = linear mixed effects model, *SE* = standard error, *df* = degrees of freedom. Subject was included as random effect. Trial, block, and treatment order were included as fixed effects. *N* = 60. Subgroup was defined as factor with levels males from previous study, females from previous study, and females from current study. | | | | | |

**Table S18**. Post-hoc *t*-tests (Tukey-adj.) for LMER models in pressure pain (VAS 70) comparing the pain ratings at HI and LI exercise for each subgroup.

|  | *T* | *SE* | *df* | *T-ratio* | *P* |
| --- | --- | --- | --- | --- | --- |
| Males (previous study) | -0.57 | 2.12 | 574 | -0.27 | 0.79 |
| Females (previous study) | -1.51 | 1.95 | 574 | -0.78 | 0.44 |
| Females (current study) | 2.11 | 2.34 | 582 | 0.90 | 0.37 |
| SE = standard error, df = degrees of freedom. LI = low-intensity, HI = high-intensity. Males (previous study): *n* = 18; females (previous study): *n* = 21; females (current study): *n* = 21. | | | | | |

**Table S19.** Full linear model output from the model including FTP on difference score heat pain ratings (LI – HI exercise) in the pooled samples.

| Fixed Effects | Estimate | *SE* | *t* | *P* |  |
| --- | --- | --- | --- | --- | --- |
| Intercept | -8.34 | 3.84 | -2.17 | **0.03** |  |
| FTP | 4.99 | 2.05 | 2.43 | **0.02** |  |
| Treatment_order | -2.15 | 2.13 | -1.01 | 0.32 |  |
| FTP = functional threshold power (weight-corrected), *SE* = standard error, *df* = degrees of freedom. | | | | | |

**Table S20.** Full linear model output from the model including FTP and sex on difference score heat pain ratings (LI – HI exercise) in the pooled samples.

| Fixed Effects | Estimate | *SE* | *t* | *P* |  |
| --- | --- | --- | --- | --- | --- |
| Intercept | -3.99 | 4.09 | -0.98 | 0.33 |  |
| FTP | 3.05 | 2.27 | 1.35 | 0.18 |  |
| Sex | -24.36 | 9.57 | -2.55 | **0.01** |  |
| Treatment_order | -2.27 | 2.18 | -1.04 | 0.30 |  |
| FTP x Sex | 11.12 | 4.69 | 2.37 | **0.02** |  |
| FTP = functional threshold power (weight-corrected), *SE* = standard error, *df* = degrees of freedom. | | | | | |

**Table S21.** Full linear model output from the model including FTP and subgroup on difference score heat pain ratings (LI – HI exercise) in the pooled samples.

| Fixed Effects | Estimate | *SE* | *t* | *P* |  |
| --- | --- | --- | --- | --- | --- |
| Intercept | -46.10 | 12.70 | -3.63 | **0.0006** |  |
| FTP | 21.65 | 6.08 | 3.56 | **0.0008** |  |
| Subgroup | 19.01 | 6.04 | 3.15 | **0.003** |  |
| Treatment_order | -4.00 | 2.22 | -1.80 | 0.08 |  |
| FTP x Subgroup | -7.99 | 2.76 | -2.89 | **0.005** |  |
| FTP = functional threshold power (weight-corrected), *SE* = standard error, *df* = degrees of freedom. Subgroup was defined as factor with levels males from previous study, females from previous study, and females from current study. | | | | | |

**Table S22.** Full linear model output from the model including FTP on difference score pressure pain ratings (LI – HI exercise) in the pooled samples.

| Fixed Effects | Estimate | *SE* | *t* | *P* |  |
| --- | --- | --- | --- | --- | --- |
| Intercept | -2.68 | 5.21 | -0.51 | 0.61 |  |
| FTP | 0.57 | 2.78 | 0.21 | 0.84 |  |
| Treatment_order | 3.08 | 2.92 | 1.06 | 0.30 |  |
| FTP = functional threshold power (weight-corrected), *SE* = standard error, *df* = degrees of freedom. | | | | | |

**Table S23.** Full linear model output from the model including FTP and sex on difference score pressure pain ratings (LI – HI exercise) in the pooled samples.

| Fixed Effects | Estimate | *SE* | *t* | *P* |  |
| --- | --- | --- | --- | --- | --- |
| Intercept | -0.87 | 5.84 | -0.15 | 0.88 |  |
| FTP | -0.41 | 3.23 | -0.13 | 0.90 |  |
| Sex | -9.91 | 13.78 | -0.72 | 0.48 |  |
| Treatment_order | 3.27 | 3.14 | 1.04 | 0.30 |  |
| FTP x Sex | 4.85 | 6.74 | 0.72 | 0.48 |  |
| FTP = functional threshold power (weight-corrected), *SE* = standard error, *df* = degrees of freedom. | | | | | |

**Table S24.** Full linear model output from the model including FTP and subgroup on difference score pressure pain ratings (LI – HI exercise) in the pooled samples.

| Fixed Effects | Estimate | *SE* | *t* | *P* |  |
| --- | --- | --- | --- | --- | --- |
| Intercept | -28.71 | 18.61 | -1.54 | 0.13 |  |
| FTP | 12.71 | 8.90 | 1.43 | 0.16 |  |
| Subgroup | 12.83 | 8.12 | 1.46 | 0.15 |  |
| Treatment_order | 2.48 | 3.26 | 0.76 | 0.45 |  |
| FTP x Subgroup | -5.78 | 4.02 | -1.44 | 0.16 |  |
| FTP = functional threshold power (weight-corrected), *SE* = standard error, *df* = degrees of freedom. Subgroup was defined as factor with levels males from previous study, females from previous study, and females from current study. | | | | | |

**Table S25.** Full linear model output from the model including Training Volume on difference score heat pain ratings (LI – HI exercise) in the pooled samples.

| Fixed Effects | Estimate | *SE* | *t* | *P* |  |
| --- | --- | --- | --- | --- | --- |
| Intercept | -0.24 | 1.73 | -0.14 | 0.89 |  |
| Training Volume | 0.10 | 0.12 | 0.78 | 0.44 |  |
| Treatment_order | -0.75 | 2.16 | -0.35 | 0.73 |  |
| Training Volume measured as training hours per week. *SE* = standard error, *df* = degrees of freedom. | | | | | |

**Table S26.** Full linear model output from the model including FTP and sex on difference score heat pain ratings (LI – HI exercise) in the pooled samples.

| Fixed Effects | Estimate | *SE* | *t* | *P* |  |
| --- | --- | --- | --- | --- | --- |
| Intercept | -0.45 | 2.00 | -0.23 | 0.82 |  |
| Training Volume | 0.16 | 0.12 | 1.30 | 0.20 |  |
| Sex | 5.33 | 3.40 | 1.57 | 0.12 |  |
| Treatment_order | -0.92 | 2.15 | -0.42 | 0.67 |  |
| Training Volume x Sex | -1.14 | 0.49 | -2.33 | **0.02** |  |
| Training Volume measured as training hours per week. *SE* = standard error, *df* = degrees of freedom. | | | | | |

**Table S27.** Full linear model output from the model including FTP and subgroup on difference score heat pain ratings (LI – HI exercise) in the pooled samples.

| Fixed Effects | Estimate | *SE* | *t* | *P* |  |
| --- | --- | --- | --- | --- | --- |
| Intercept | 2.69 | 4.48 | 0.60 | 0.55 |  |
| Training Volume | -0.99 | 0.69 | -1.44 | 0.16 |  |
| Subgroup | -0.43 | 2.05 | -0.21 | 0.83 |  |
| Treatment_order | -1.67 | 2.34 | -0.71 | 0.48 |  |
| Training Volume x Subgroup | 0.37 | 0.24 | 1.54 | 0.13 |  |
| Training Volume measured as training hours per week. *SE* = standard error, *df* = degrees of freedom. Subgroup was defined as factor with levels males from previous study, females from previous study, and females from current study. | | | | | |

**Table S28.** Full linear model output from the model including FTP on difference score pressure pain ratings (LI – HI exercise) in the pooled samples.

| Fixed Effects | Estimate | *SE* | *t* | *P* |  |
| --- | --- | --- | --- | --- | --- |
| Intercept | -1.09 | 2.24 | -0.49 | 0.63 |  |
| Training Volume | -0.14 | 0.16 | -0.92 | 0.36 |  |
| Treatment_order | 4.04 | 2.81 | 1.44 | 0.16 |  |
| Training Volume measured as training hours per week. *SE* = standard error, *df* = degrees of freedom. | | | | | |

**Table S29.** Full linear model output from the model including FTP and sex on difference score pressure pain ratings (LI – HI exercise) in the pooled samples.

| Fixed Effects | Estimate | *SE* | *t* | *P* |  |
| --- | --- | --- | --- | --- | --- |
| Intercept | -0.78 | 2.72 | -0.29 | 0.77 |  |
| Training Volume | -0.16 | 0.17 | -0.97 | 0.34 |  |
| Sex | -1.64 | 4.63 | -0.35 | 0.73 |  |
| Treatment_order | 3.96 | 2.92 | 1.35 | 0.18 |  |
| Training Volume x Sex | 0.23 | 0.67 | 0.35 | 0.73 |  |
| Training Volume measured as training hours per week. *SE* = standard error, *df* = degrees of freedom. | | | | | |

**Table S30.** Full linear model output from the model including FTP and subgroup on difference score pressure pain ratings (LI – HI exercise) in the pooled samples.

| Fixed Effects | Estimate | *SE* | *t* | *P* |  |
| --- | --- | --- | --- | --- | --- |
| Intercept | -5.65 | 5.99 | -0.94 | 0.35 |  |
| Training Volume | 0.41 | 0.92 | 0.45 | 0.66 |  |
| Subgroup | 2.72 | 2.74 | 0.83 | 0.41 |  |
| Treatment_order | 3.21 | 3.13 | 1.03 | 0.31 |  |
| Training Volume x Subgroup | -0.21 | 0.32 | -0.64 | 0.52 |  |
| Training Volume measured as training hours per week. *SE* = standard error, *df* = degrees of freedom. Subgroup was defined as factor with levels males from previous study, females from previous study, and females from current study. | | | | | |
