# Supplementary material for: Fitness level, but not sex, affects exercise-induced pain modulation
Source: eLife. 2026 Oct 30;15:RP109061. doi: 10.7554/eLife.109061 (PMC13722413; doi:10.7554/eLife.109061)
Supplement: Peer review history

# Editor report: eLife Assessment

**DOI:** 10.7554/eLife.109061.3.sa0

**Authors:** José Biurrun Manresa

**Affiliations:** National Scientific and Technical Research Council (CONICET), National University of Entre Ríos (UNER) Argentina

**Keywords:** Incomplete, Useful

This study proposes that fitness level, but not sex, influences exercise-induced hypoalgesia in women. However, the evidence to support this claim is **incomplete**: the conclusions rely on a small-effect interaction that emerges only under specific conditions, the findings are inconsistent across pain modalities and stimulus intensities, and the absence of a baseline condition limits the interpretability of results as reflecting true hypoalgesia. Additionally, the methods by which fitness level was categorized across cohorts can be questioned. The results of this study could be **useful** to improve future experimental designs exploring these factors in more depth.

# Referee report: Reviewer #2 (Public review)

**DOI:** 10.7554/eLife.109061.3.sa1

I appreciate the substantial amount of work the authors have invested in revising the manuscript. The response is extensive, and the addition of pooled frequentist and Bayesian analyses represents a considerable and technically sophisticated effort to address the concerns raised during review. The authors have also improved the use and presentation of the continuous pain-rating data and have responded constructively to several methodological points.

Nevertheless, with respect to my original review, I do not think the revisions have yet aligned the manuscript's central claims with the specificity and uncertainty of the evidence. The additional analyses provide some evidence consistent with a contribution of fitness to heat-pain modulation, but they cannot fully resolve the limited internal replication, selected sample or differences in measurement procedures between studies. Nor do they establish that sex has no effect on pain modulation. In my view, the evidence supports a modest, exploratory conclusion that fitness may contribute to variability in heat-pain modulation under specific conditions, rather than the more categorical conclusion that exercise-induced pain modulation depends on fitness but not sex. The manuscript would therefore be strengthened by reflecting this distinction more consistently in the title, abstract, Discussion and Conclusion. My remaining concerns, organised in relation to the original review, are detailed below.

(1) The core interpretation may not be fully supported by the data

(1.1) Lack of baseline condition

I am satisfied with the authors' substantive response to Comment 1.1, however I note that the authors are still attributing their findings to "hypoalgesia" in the Discussion.

(1.2) Lack of internal replication across conditions

The pooled analyses do not resolve the original lack of internal replication. The substantive effect remains largely restricted to heat pain at the highest stimulus intensity and is not clearly reproduced across stimulus intensities or for pressure pain. The authors' response describes the evidence more cautiously, stating that fitness "contributes to variability in exercise-induced pain modulation". However, the title and principal conclusions do not reflect the exploratory, modality-specific and statistically uncertain nature of the evidence.

(1.3) Deviations from the original study and selective use of data

I appreciate the addition of analyses using the continuous pain ratings, which addresses my concern that the richness of the data from the current study was not being fully used. However, the decision to pool the current and previous datasets introduces a related and potentially more consequential comparability problem. The previous study used retrospective post-stimulus ratings, whereas the current study used continuous ratings from which peak values were extracted. Rescaling these measures to a common numerical range does not establish their measurement equivalence. This is particularly important because study membership is closely confounded with the participant characteristics central to the manuscript: the higher-fitness female group was recruited and assessed in the current study, whereas the comparison groups were assessed using the earlier procedure. Consequently, apparent effects of fitness may partly reflect differences in outcome acquisition between studies. Indeed, including study membership improves model fit substantially more than including fitness.

(1.4) Over-reliance on null results regarding sex differences

I do not think the new Bayesian analyses resolve my original concern regarding the interpretation of the null sex effect. The authors state that the analyses provide no evidence for a moderating role of sex, but they do not report a Bayes factor clearly supporting the null hypothesis for the exercise intensity × sex interaction. In fact, Figure 5 clearly shows that the association between fitness and pain modulation is significant in males but not females. Panels D-F indicate uncertainty regarding a differently specified exercise intensity × sex interaction, rather than evidence for its absence. The figure therefore suggests that the role of sex remains unresolved and may qualify the association attributed to fitness. The authors should therefore distinguish absence of evidence from evidence of absence and temper the conclusion that exercise-induced pain modulation depends on fitness "but not sex".

(2) Limited sample and lack of diversity

The manuscript does not acknowledge the limited generalisability of this small, highly selected sample of physically active women recruited primarily from cycling clubs. Although the rebuttal states that the findings should be interpreted within the context of the sample studied, this qualification is absent from the Limitations section. It should be added, and the conclusions restricted accordingly.

(3) Methodological choices limit interpretability of the data

(3.1) The use of a non-standard VAS scale (0-150 with a fixed pain threshold at 50) is unconventional and may influence how participants report pain, while limiting comparability with related literature.

The authors' response explains the rationale for the modified scale but does not fully address its implications for measurement comparability. In particular, setting all ratings below 50 to zero is not merely rescaling: it collapses distinct responses into a single value and may alter the distribution of the outcome. The cited study addresses the relationship between real-time and retrospective ratings, but does not validate this scale or transformation.

# Author comment: Author response

**DOI:** 10.7554/eLife.109061.3.sa2

**Authors:** Janne Ina Nold, Tahmine Fadai, Zora Pia Gerbers, Christian Büchel

**Affiliations:** University Medical Center Hamburg-Eppendorf Hamburg Germany; University Medical Center Hamburg-Eppendorf Hamburg Germany; University Medical Center Hamburg-Eppendorf Hamburg Germany; University Medical Center Hamburg-Eppendorf Hamburg Germany

The following is the authors’ response to the original reviews.

**Public Reviews:**

**Reviewer #1 (Public review):**

Summary:

The current study is a follow-up to a previously published study by the same research group (Nold et al. 2025). In the previous study, the authors had included a set of exploratory analyses which assessed the effects of fitness level (denominated by a relative FTP), sex, and drug treatment (Naxolone versus placebo). In this previous study, the authors state that "exploratory analysis showed a significant main effect of fitness level on differences in pain ratings in the [saline] condition... suggesting increased hypoalgesia with increasing fitness levels, pooled across all stimulus intensities".

In the current study, the authors have recruited an additional 22 female participants (21 included in analysis) from local cycling clubs to assess if fitness level does indeed impact exercise-induced hypoalgesia responses to experimental thermal and pressure pain models.

Strengths:

The current study has the potential to present a convincing argument about the effect of fitness level and potentially other factors (e.g., sex) on exercise-induced hypoalgesia responses. Combining data across two of their primary studies would be highly fruitful to the research community interested in this area. Specifically, it has the potential to inform sports medicine practitioners and how they administer exercise protocols to help those experiencing pain with a further consideration for the fitness level (and maybe sex) of their patients.

Weaknesses:

However, the current study makes several bold claims about the role of fitness level and sex on exercise-induced hypoalgesia, which I do not believe that this study on its own - or in conjunction with the previously published study by the same authors - can make at present. Namely, the current study does not appear to conduct any specific analyses between the cohorts from either study (current and present). The results mention a difference in the group mean values in "fitness level" between cohorts, but the analysis itself on pain responses/exercise-induced hypoalgesia is limited only to the cohort from the current study. If the authors wanted to provide a convincing argument that fitness level has an effect on exercise-induced hypoalgesia, then the analysis of this study would have to include an analysis between the groups considered to be of "high" and "low" fitness level. I do not think the current study does this. Instead, it makes an assumption from the previous study (Nold et al. 2025) which only states that "exploratory analysis showed a significant main effect of fitness level on differences in pain ratings in the [saline] condition... suggesting increased hypoalgesia with increasing fitness levels, pooled across all stimulus intensities". The analysis of this study would have to include fitness level "high fitness" versus "low fitness" of participants across both studies in its statistical model to properly discern if fitness level has an impact on exercise-induced hypoalgesia.

A similar comment can be made with respect to sex differences, as these have not been assessed in the analysis of this study either.

Another area of weakness in this study is how "fitness level" has been demarcated across participants. One issue is how authors have assumed that the current cohort is 'fit', whereas the previous cohort was 'less fit', meaning that the authors could be coming to false conclusions about fitness level. In detail, figures within the current study show a large overlap between the 'fit' and 'less fit' cohorts, where some participants have a higher relative functional threshold power (FTP) in the 'less fit' cohort than the 'fit' cohort and vice versa. Therefore, I believe the authors should better demarcate between those that are in the 'more fit' and 'less fit' groups according to a validated and well-established criterion from the kinesiology and sport science literature. That being said, I think this may be problematic in some ways as FTP is considered a relatively poor measure to denote fitness levels, a limitation highlighted in the previous study's review.

Altogether, whilst I commend the researchers on their body of work across the two studies, the current methods and analysis provide an incomplete assessment of their primary research question, and therefore, I would urge the authors to reconsider some of their methods/analysis and the framing of their results to better reflect the main research question they have attempted to answer. Likewise, I would recommend that readers ensure they consider the current results with caution until the authors have addressed some areas of concern which currently limit their main conclusions.

We sincerely thank the reviewer for their thorough and insightful comments and for the time invested in reviewing our work. In response, we have made several important revisions, particularly to address the major concerns regarding fitness level and sex differences.

Specifically, we have:

(1) Conducted additional pooled analyses to directly evaluate fitness and sex effects.

We pooled the data from the previous and current studies and conducted linear mixed-effects models investigating the effect of exercise intensity between the subgroups, as well as specifically testing the effect of fitness level and sex across the pooled data. This allowed us to directly test the effects of fitness level and sex on a common scale rather than infer these effects from separate cohorts. To quantify evidence both for and against the presence of exercise-induced pain modulation and its moderation by fitness, sex, and study sample, we estimated Bayes factors comparing nested models of increasing complexity. In addition, we examined the posterior distributions of the regression coefficients (β) and subgroup- specific (males from previous study, females from previous study, females from current study) exercise effects to estimate the magnitude and uncertainty of these effects. The Bayesian analyses provided some evidence that fitness level moderated exercise-induced pain modulation, but sex did not. Importantly, these findings were consistent with the overall pattern of results obtained in the frequentist analyses. All corresponding statistics in the main manuscript and Supplementary Materials have been updated accordingly. To improve transparency, the pooled analyses are now presented in the dedicated Results subsections and accompanied by new figures (Figures 4 and 5). Furthermore, we have adapted the Title, Discussion and Conclusion to more closely reflect the results.

(2) Clarified the assessment of fitness level.

We expanded our consideration of fitness level and training status by discussing training volume as an additional indicator of training status. We also acknowledge that fitness is a continuous construct and that some overlap in FTP values exists between cohorts. To address this concern, we conducted pooled analyses using FTP as a continuous predictor rather than relying solely on cohort membership. For consistency and comparability with the previous study, FTP remained our primary indicator of fitness level, while analyses using training volume are now reported in the Supplementary Materials.

(3) Expanded the mechanistic background on the effect of fitness level and sex on EIH.

We expanded the mechanistic background in both the Introduction and Discussion to provide a clearer theoretical rationale for how fitness level and sex may influence exercise-induced hypoalgesia (EIH), drawing on relevant experimental and clinical literature.

**Reviewer #2 (Public review):**

This study addresses an important question regarding exercise-induced modulation of pain in women, but the conclusions appear to be based on relatively limited and selective evidence. The authors report an interaction between exercise intensity and stimulus intensity, which they interpret as evidence for exercise-induced hypoalgesia and conclude that fitness, but not sex, modulates this effect. However, this main result relies on a relatively small interaction that emerges only under specific conditions, with inconsistent findings across pain modalities and stimulus intensities, and an analysis approach that does not fully exploit the continuous pain ratings collected. The lack of a baseline condition further limits the interpretability of the findings as reflecting hypoalgesia, and overall, the data provide a rather constrained basis for drawing broader conclusions.

Strengths:

(1) The focus on women is important and timely, particularly given the ambiguity in prior findings and the historical bias toward male-dominated samples.

(2) The attempt to revisit previous findings in a new cohort is valuable in principle.

Weaknesses:

(1) The core interpretation may not be fully supported by the data

The central claim-that the results demonstrate exercise-induced hypoalgesia and its dependence on fitness but not sex-does not appear to be fully supported by the evidence presented.

(1.1) Lack of baseline condition

The absence of a no-exercise baseline substantially limits interpretation. The study compares high- and low-intensity exercise, but without a baseline, it is not possible to determine whether either condition produces hypoalgesia or hyperalgesia relative to calibration. The observed HI-LI difference, therefore, reflects only a relative contrast between exercise intensities, not an absolute reduction in pain. As a result, attributing the findings to "hypoalgesia" may be difficult to justify fully.

(1.2) Lack of internal replication across conditions

The reported effect is highly specific and does not clearly generalise across the experimental design. It emerges significantly only for heat pain at the highest stimulus intensity, with no clear effects for other intensities and for pressure pain. Moreover, the main statistical result is a relatively small interaction effect with a modest p value, which translates into a difference of approximately 6-8 VAS units on a 150 scale. This combination-a small effect size, limited statistical strength, and restriction to a single condition-substantially weakens the evidence for a robust or generalisable effect.

(1.3) Deviations from the original study and selective use of data

Although framed as a follow-up to previous work, the current study introduces substantial methodological changes, particularly in the acquisition and scaling of pain ratings (continuous vs post-hoc ratings, modified VAS with sub-threshold range). Despite collecting rich continuous data, the analysis focuses on peak responses to approximate the previous study. While this may aid comparability, it results in a strong emphasis on a single data point (highest intensity), rather than leveraging the full dataset. This limits both interpretability and comparability.

(1.4) Over-reliance on null results regarding sex differences

The conclusion that fitness, but not sex, modulates exercise-induced pain may not be directly supported by the data presented. The current study includes only highly fit women, and comparisons with men or less-fit women rely on non-significant differences in a previous cohort. The absence of a significant difference does not provide evidence for equivalence, and no formal statistical support for a null effect is provided. As such, conclusions about the absence of sex differences would unfortunately benefit from more cautious interpretation.

(2) Limited sample and lack of diversity

The dataset is narrow in scope, comprising a small sample (N = 21) of healthy, highly fit women. Key demographic characteristics (e.g. age range, BMI distribution) are not fully presented, explored or discussed. This limits generalisability and makes it difficult to draw broader conclusions about exercise-induced pain modulation in women, as the main focus of the study.

(3) Methodological choices limit the interpretability of the data

Several methodological decisions would benefit from stronger justification:

(3.1) The use of a non-standard VAS scale (0-150 with a fixed pain threshold at 50) is unconventional and may influence how participants report pain, while limiting comparability with related literature.

(3.2) Participants explicitly reported expecting exercise to reduce pain, introducing a potential confound that is not presently addressed.

(3.3) A more comprehensive use of the full time series of pain ratings would provide a stronger and more transparent basis for interpretation of the present findings.

We sincerely thank the reviewer for their time and effort invested in reviewing our manuscript and appreciate the comments. In response to the points raised by the reviewer, we have now updated our work. Specifically, we have:

(1) Pooled samples and strengthened inferential framework:

We pooled the current and previous datasets to provide a stronger basis for the primary interpretation of the findings. On this combined dataset, we conducted both frequentist linear mixed-effects models and complementary Bayesian analyses, allowing us to directly test effects of fitness level, sex, and exercise intensity within a unified framework and to quantify both effect sizes and evidence for/against key hypotheses. The Title, Results, Methods and Discussion have been adapted to more closely reflect the results.

(2) Addressed sample limitations through pooled analysis:

To mitigate limitations related to sample size and composition in the individual studies, we extended all primary inferential analyses to the pooled dataset. This approach increases statistical power, improves the stability of estimates, and enables a more reliable examination of between-group effects across fitness level and sex, thereby strengthening the generalisability of the findings.

(3) Expanded analyses of continuous pain ratings:

To address methodological concerns regarding the use of a summary pain metric, we supplemented the primary peak-based analyses with additional linear mixed-effects models on the full time-series data. Specifically, we modelled time × exercise intensity interactions for both heat and pressure pain, thereby capturing the dynamic structure of pain responses and ensuring that conclusions are supported by the richness of the continuous rating data. These analyses have been added to the Supplementary Materials.

**Recommendations for the authors:**

**Reviewer #1 (Recommendations for the authors):**

Synopsis

The current study is a follow-up to a previously published study by the same research group (Nold et al. 2025). In the previous study, the authors had included a set of exploratory analyses which assessed the effects of fitness level (denominated by a relative FTP), sex, and drug treatment (Naxolone versus placebo).

In the current study, the authors have recruited an additional 22 female participants (21 included in analysis) from local cycling clubs to assess if fitness level impacts exercise-induced hypoalgesia responses to experimental thermal and pressure pain models.

All the analysis of this study seems to be specific to the "fit" cohort from this study. No analysis or figures seem to show differences between cohorts from the current and previous studies. For this study, the authors found that maximum pain ratings after application of a thermal stimulus and pressure pain stimulus were lower after a high-intensity exercise compared to a lower-intensity exercise. There was also an exercise intensity × stimulus intensity interaction when thermal stimulations were applied, suggesting that the exercise-induced hypoalgesia response varies across exercise intensities as the stimulus intensity increases/decreases. The same cannot be said for pressure pain according the statistical analysis outputs of this study, although inspection of Figure 3B (top row) does illustrate that at higher stimulus intensities, there is a difference between exercise intensities for maximal pain ratings, whereas there is no difference at lower stimulus intensities (30 and 50 VAS). However, observing the changes in maximum pain ratings (Figure 3C) does show similar changes across all stimulus intensities for pressure pain, unlike thermal pain. Therefore, the authors claim that there is an effect of fitness level on the exercise-induced hypoalgesia response.

Overall, the authors have completed a neat supplementary study to their previous study. However, there are several areas that I believe should be addressed within the current manuscript.

First, the authors regularly state "...the hypoalgesic response to exercise depends on fitness level but not on sex". As mentioned earlier, the results section of the previous study states that "exploratory analysis showed a significant main effect of fitness level on differences in pain ratings in the [saline] condition... suggesting increased hypoalgesia with increasing fitness levels, pooled across all stimulus intensities". In this study, the authors do not seem to have completed a direct comparison between the two study cohorts. The results for the current study only seem to discuss the effects of exercise intensity within this study cohort. If this study is to be treated as a stand-alone study, then the data that is presented should make it clear that the effect of fitness level is being assessed. Currently, the results and the figures only illustrate the effects of exercise intensity and stimulus intensity, not fitness level (or sex), which are mentioned in the title, abstract, and most discussions.

Second, on the topic of sex-related effects, as authors repeatedly state ""...the hypoalgesic response to exercise depends on fitness level but not on sex", I think it is worth noting that this statement would require an a priori assessment of sex differences on exercise-induced hypoalgesia responses. As the previous study (exploratory) and this study (no sex difference analysis) do not include this a priori assessment, I do not think they can conclude that sex is not a factor in the exercise-induced hypoalgesia response, though they can surmise that fitness level may have an influencing role if it was specifically tested in this study. If the authors wish to judge the role of sex, there ought to analysis on this specifically.

Third, whilst the authors have tested the effects of "fitness level", I urge the authors to consider how they have demarcated between fitness levels in this study and any other (validated) criterion that could be used in place of their current method to distinguish between "fit" and "less fit" participants. To explain the cohort of the current study is judged to be "fitter" than the previous study based on the group mean relative FTP. Yet, observing Figure 1, there are several participants with lower relative FTP in the "fit" cohort than the "less fit" cohort, and vice versa. I would recommend the authors to consult with some kinesiologists/sport scientists about this issue. As the central topic of this study is the effect of fitness level, this warrants careful consideration, as the current method to split the groups is problematic, especially as there is clear overlap between the two groups in the metric that the authors have used (relative FTP).

Fourth, I think it would be highly beneficial for the authors to add more discussion around the theoretical and mechanistic accounts about why fitness level (and sex) would impact exerciseinduced hypoalgesia responses. The current manuscript provides a nice account of the current literature in terms of the direction of each study's results, yet they do not explore why fitness level or sex has such effects. This would be highly valuable to a reader to set the tone of the study rationale in the introduction and provide more depth to the authors' discussion of their results.

Below I have highlighted all my comments that have been separated into MAJOR and MINOR comment sections.

I hope my comments are of use to the authors, and I would like to thank them and the journal for the opportunity to review their paper on a truly interesting and insightful topic.

We thank the reviewer for their insightful comments and appreciate the time invested in reviewing our work. In the following, we have addressed all comments raised by the reviewer and marked all changes in the manuscript.

Major Comments

(1) In my opinion, it would enhance the manuscript if the authors provided a theoretical or mechanistic account of why fitness levels or sex would impact the exercise-related hypoalgesia effects. Though some reasoning about the mechanisms of fitness level and sex is mentioned in the discussion of the previous study (Nold et al. 2025), and even if the overall research findings are mixed at present, I do not think it would be overkill to provide the rationale for why the authors do/do not predict a certain response in the current study after their analyses. On this note, the authors may argue that the manuscript content from L52-61 is an account of the previous literature. However, whilst this literature sets out the directions of previous research findings, as I read this, I do not think there is a theoretical framework to underpin why fitness level or sex would impact exercise-induced hypoalgesia (EIH). References already included in the manuscript should have some discussions around this (e.g., Sluka et al. 2018; Koltyn 2000; Koltyn et al. 2014).

We thank the reviewer for their comment. We agree that the mechanistic rationale for why fitness level and sex may influence EIH could be described in more detail. Accordingly, we have expanded the Introduction/Discussion to provide a more comprehensive overview of the potential mechanisms underlying these effects, drawing on previous EIH literature (e.g., Sluka et al., 2018; Koltyn, 2000; Koltyn et al., 2014) as well as evidence relating to changes in endogenous modulatory systems due to repeated exercise (Anderson & Shivakumar, 2013; Gajic et al., 2026; Matei et al., 2023; Saanijoki et al., 2022; Song et al., 2022) as well as sex differences in these neuromodulatory systems potentially contributing to sex differences in EIH (Blanton et al., 2021; Castelli et al., 2014; Fullerton et al., 2018; Levine et al., 2021; Loyd et al., 2008; Zubieta et al., 2002).

(2) The study sets out an aim, but I do not see any (a priori) hypotheses in the intro or methods. If no hypotheses were registered, this can be reported in the discussion and factored into the discussion about why certain results would/would not be expected.

The reviewer is correct that no formal, pre-registered hypotheses were specified for this study. As this study was designed as a follow-up to our previous study, which reported no exercise-induced pain modulation in relatively less fit females, the present investigation was primarily exploratory in nature and aimed to examine whether exercise-induced pain modulations would be observed in a sample of females with significantly higher fitness levels. We have now clarified this in the discussion and acknowledge the exploratory nature of the study when interpreting the findings.

(3) Is there a sample size justification, sensitivity analysis, or post-hoc calculation for the additional 21 participants to detect effects for fitness level and sex?

We thank the reviewer for this question. No formal a priori sample size calculation was performed for the recruitment of the additional 21 participants. The current study was designed as a follow-up to our previous work, in which female participants were, on average, slightly less fit than male participants. To better disentangle the effects of fitness and sex, we therefore aimed to recruit a comparable number of female participants with higher fitness levels. Accordingly, we targeted a sample size similar to that of the female subgroup in the previous study.

(4) I have a wider question about where there has truly been a test between fitness levels.

Definition and measurement of "fitness level".

Is there an appropriate rationale that relative FTP is an appropriate measure to distinguish between fitness levels? Understandably, this is done for consistency between the two studies, and there may be mention of the use of the FTP measure in the original study; however, it would be worth justifying the use of the relative FTP measure to categorise fitness level with previous literature to back this up.

We thank the reviewer for this very valid and important point regarding both the validity of FTP as a proxy for fitness and the distinction between fitness levels. Previous research has shown that the FTP and FTP20 tests are reliable and convenient methods to estimate approximate measures of VO2max (Denham et al., 2020) and that the FTP test is a useful test for performance prediction in moderately trained cyclists (Sørensen et al., 2019). Furthermore, we argue that FTP represents a continuous performance measure in our studies instead of a categorical variable distinguishing between fit and unfit individuals, and we have strengthened the manuscript by providing a clearer justification for its use as a field-based proxy of endurance fitness, supported by previous literature linking FTP to more direct physiological markers such as VO_2max_ (Denham et al., 2020). Since we have used FTP in the previous sample as a measure of fitness level, we maintained this for consistency in the current study.

We also acknowledge that, despite statistically significant group differences, there is meaningful overlap between the males of the previous study and the females of the current study and that “fitness level” should be interpreted as a continuum rather than a strict categorical separation. Accordingly, the labels “fit” and “unfit” should be interpreted relative to the samples included in the current and previous studies rather than as absolute classifications. To reflect this more accurately, we have revised the manuscript to refer to participants as having higher or lower fitness levels, which should be interpreted within the context of the pooled sample rather than as discrete fitness categories.

Classification of fitness level.

In L110-111 - The statistical analysis does show a difference between groups, but Figure 1A does show that there is a crossover with around half the sample from each study cohort. That is, the current study had ~16 participants who had a lower relative FTP than the highest relative FTP from the previous study. When removing the highest participant (outlier) in the previous study, there are still 7 participants (a third of the sample) with a lower relative FTP than participants considered of a 'lower' fitness level than the previous study.

Essentially, on the group level, there is a statistical difference in relative FTP, but there is notable overlap between the two groups.

We thank the reviewer for this observation. Our aim was not to create entirely non-overlapping fitness categories, but rather to recruit a female sample with significantly higher average fitness levels than the females included in the previous study. As shown in the revised manuscript, females from the current study had, on average, significantly higher relative FTP values than females from the previous study, whereas no significant difference was observed between females from the current study and males from the previous study. We also report training volume as an additional indicator of training status; however, because training volume was self-reported, we consider FTP to provide a more objective and sensitive measure of fitness. The partial overlap in FTP values between samples is not unexpected and, in fact, facilitates comparisons across studies by ensuring that the samples are not entirely distinct populations.

Could another distinction be made based on:

- A set value (e.g., 2.0 W/kg-1);

- Another value from previous literature, and the analysis be re-run with this classification of participants? The approach to use a set value is consistent with kinesiology literature that uses set values to demarcate fitness level for other more common, measures like VO2max (see ACSM guidelines and de Pauw et al. 2013);

- Or by dissociating between training volume and slightly tweaking the wording to "training status/volume" (although I am cognisant that this would introduce an incongruence in terminology between the authors' studies, but other studies that have been referenced [e.g., Geva and Defrin, do specifically test between training volume]).

We thank the reviewer for their suggestions. We would like to refrain from making another distinction based on a set value and dividing the group into “fit” and “unfit” individuals. Instead, we have pooled the samples and treated FTP as a continuous variable, which preserves statistical power and avoids the loss of information associated with dichotomising a continuous measure.

To address the reviewers' suggestion of including training volume as a measure of fitness level, we have repeated our new analyses, namely the LMER model using training volume as a predictor for difference pain ratings (LI – HI Exercise pain ratings), in the pooled sample. These analyses yielded no significant effects for training volume, the interaction of training volume x sex, or training volume x subgroup in either heat or pressure pain. Furthermore, the training volume is potentially influenced by outliers in the current study sample. For completeness, we have added these analyses and according visualisation to the Supplements (Fig. S6).

We believe that the FTP provides a more sensitive and objective measure of cardiovascular fitness level in our study, as it is not susceptible to recall bias, differences in training intensity and differences in training efficacy. We also note that other studies (Geva & Defrin, 2013) did not rely solely on self-reported training volume as a measure of fitness level, but recruited participants based on training history and athletic status (e.g. competitive triathletes vs. active controls).

For these reasons, we consider the FTP the most appropriate primary indicator of fitness level in the current (and previous) study, while still reporting training volume in the main manuscript to consider.

The above suggestions would also allow to select matched pairs from the male cohort when comparing effects between sexes. Altogether, though, I believe this comment to be the most important in my review of this paper, as the primary question depends strongly on how fitness level is defined and categorised by the researchers, and this requires careful consideration and validation from previous literature if such decisive conclusions are to be drawn from the results. Therefore, a clearer description of the reason why certain effects are expected (theory/mechanisms) and how participants have been categorised will be highly valuable for readers. It may be useful for the authors to reach out to kinesiologists/sport scientists within their peer groups to help with this comment.

To further address the concern regarding between-cohort comparability, we have now pooled the samples from the previous and current study and re-analysed the data fitness level as a continuous variable rather than relying on cohort-based comparisons. Specifically, we conducted regression analyses examining fitness level, sex, and their interaction, as well as subgroup analyses including males from the previous study, females from the previous study, and females from the current study. These analyses were complemented by Bayesian model comparison and posterior inference. The results are now reported in the Results section. We also acknowledge that fitness is best viewed as a continuum rather than a discrete characteristic and have revised the manuscript accordingly, replacing references to “fit” and “unfit” participants with descriptions of relatively higher and lower fitness levels within the pooled sample.

Finally, as mentioned in the previous comment (major comment #1), we have expanded the Introduction and Discussion to more clearly outline the mechanistic and theoretical rationale for expecting effects of fitness level and sex on exercise-induced pain modulation, drawing on relevant literature (see major comment #1). We believe these additions substantially strengthen both the conceptual justification and the analytical robustness of the manuscript.

(5) The abstract states that "...further investigation to determine if and to what extent exercise induced hypoalgesia depends on fitness level and/or sex." However, I cannot see any analysis relating to sex in the results of this study that takes the female sample of this study and the male sample of the previous study and then carries out another LNER model? I also see no report of results about sex in any of the figures/tables. If sex is part of the abstract and main research question, then I would argue it ought to be in the results and feature in figures within the main manuscript, not supplementary material.

We agree with the reviewer that, given the emphasis on fitness and sex in the Abstract and research aims, analyses directly examining sex effects should be presented in the main manuscript. In response, we pooled the samples from the previous and current studies and repeated the linear mixed-effects regression analyses used in the previous study to examine the effects of fitness and sex on exercise-induced pain modulation. We further complemented these analyses with Bayesian model comparison and parameter estimation to assess the moderating effects of fitness level and sex in the pooled dataset. The results of these pooled analyses are now reported in the corresponding Results section and are presented in the main manuscript as Figure 5. The Abstract, Results, Discussion, and Conclusions have been revised accordingly.

(6) The current abstract and main manuscript make it seem that this study completed an additional analysis on the effect of sex, but this does not appear to be the case. Statements like "together with our previous findings, this suggests that exercise-induced hypoalgesia depends on fitness level but not on sex" are quite decisive, although it does not seem that any analysis was completed between sexes in an a priori fashion. The only data I can see that assesses between sex effects is an exploratory analysis from the previous study, which was part of a larger exploration of sex - fitness level - and drug interactions.

Therefore could the authors either:

- Run this analysis between sexes;

- Or make it explicit that this study did not conduct any specific a priori analysis on sex differences in EIH at high and low intensities.

- And/or temper how decisive their conclusions on the (null) effects for sex, unless there are statistical analyses to support this.

We thank the reviewer for this suggestion. We agree that the manuscript did not include a dedicated analysis of sex effects in the combined dataset. In response, we pooled the samples from the previous and current studies and conducted additional frequentist (LMER) and Bayesian (Bayes Factor and posterior distributions) analyses examining the effects of fitness and sex on exercise-induced pain modulation. The frequentist analyses indicated an association between fitness and EIH, but not sex, while the Bayesian analyses similarly provided little evidence for sex-dependent effects. These new analyses have been added to the manuscript, and the Abstract, Results, Discussion, and Conclusions have been revised accordingly.

Section-specific Comments:

(1) Abstract

L38-40: See major comment #6 above.

As described above, we have now conducted additional analyses on the moderating effect of fitness level and sex using frequentist and Bayesian analyses and adapted the Abstract accordingly.

(2) Introduction

L68 - What is meant by "robust" in this sentence? Robust to describe the direction and magnitude of the differences in effects between sexes seems a little misrepresentative.

We thank the reviewer for their comment and agree that the wording is vague. To emphasise clarity, we have rephrased this to “[…] whereas others (Koltyn, 2000; Sternberg et al., 2001; Vaegter et al., 2014) suggest that hypoalgesia following exercise is more pronounced in females, but overall results remain inconclusive (Rice et al., 2019).”

(3) Results

L124 - What is the online rating scale? It is the word "online" in particular that is confusing here, as it is described in the methods, but just seems to be a standard handheld device with a cursor.

We thank the reviewer for their comment. In this case, “online” refers to the continuous ratings whilst the stimulus is ongoing, using a handheld rating device and using button presses. We clarified this further at the beginning of the Results and Methods sections to include “real-time (continuous) pain ratings” to avoid confusion.

L136 - I think there is a missing word here before the set of parentheses. RPE?

We thank the reviewer for detecting this and have added “RPE” before the parentheses.

L136-138 - I would amend to read something like... "This confirms the exercise intensity protocols elicited a different output, relative power, physiological demand (HR), and perceived demand (RPE)", just to be clear on what the differences have been physiologically and psychologically for the participants when exercising at the two intensities.

We agree with the reviewer and have rephrased the sentence according to their suggestion for clarity.

(4) Discussion

L190 - "results suggest that fit females show a hypoalgesic response to HI compared to LI exercise". I think there is a word missing in this sentence to make it clear what the results are.

We have revised the sentence in accordance with the new (pooled) analysis results.

L250-253 - The authors have given a fantastic account of the previous literature and the trends that each study has observed. However, in line with major comment #1, could the authors include some discussion to speculate why there are expected differences between training level/fitness level and sex (plus interactions)? Some studies that are referenced are theoretical papers, and even if the current literature is unsure about the likely mechanisms behind fitness and sex differences in EIH responses, stating this would also be useful to a reader but the authors could signpost them to specific references that would broaden their understanding after reading this study.

We have now provided a more detailed account of the potential mechanistic background on why fitness level and/or sex could influence exercise-induced modulation in the Discussion (see major comment #1).

(5) Limitations

L258-259 - If I have understood this sentence correctly and the content in L301-304, the heat stimulus (temperature of the thermal probe) that was applied to induce a 30 VAS pain intensity rating at rest actually induced no pain at all, as 30 VAS is considered below the pain threshold of "minimally painful". In retrospect, this seems like an odd choice of measurement.

It means that 30 VAS is simply a measure of warm (non-painful but detecting heat) and perturbation to the skin (pressure but not hurting) in the heat and pressure pain conditions, respectively. Therefore, to include the 30 VAS values in the analysis to dissociate whether HIGH or LIGHT intensities, fitness level, or sex have an impact on EIH seems misplaced, as there is no actual pain for which a hypoalgesia response can be tested at this intensity.

Making this clearer in the results or discussion would be worthwhile for the readers, in my opinion.

We thank the reviewer for highlighting this. In both the previous and current studies, participants were calibrated using the same procedure and rating scale (0 – 100) to identify painful stimulus intensities corresponding to VAS 30, 50, and 70. On the main experimental day, participants in the current study rated on a modified scale ranging from 0 to 150, with 50 marking the pain threshold, such that ratings below 50 reflected a non-painful rating. Importantly, the calibrated VAS 30, 50, and 70 stimuli refer to the target pain intensities established during the calibration procedure and not to the numerical values displayed on the experimental rating scale. Participants were familiarised with the scale and anchor points prior to testing and viewed only the visual analogue scale rather than numerical values during the experiment. We have adapted the wording, referring to VAS 30, 50, and 70 as intensities and not numerical ratings.

(6) Conclusion

L276-277 - Similar mention as before, can the authors please make it clear if there is a sex effect in their analyses or how this has properly been tested in a prior study through an a priori assessment to be certain that "fitness level rather than sex plays a central role in modulating EIH".

We have now tested the effect of sex in frequentist and Bayesian analyses in the pooled samples and revised the conclusion in accordance with the results.

(7) Methods

L284 - What denotes an individual being "physically active"?

We thank the reviewer for this question. “Physically active” was defined based on training volume, operationalised as regular participation in structured cycling training sessions (i.e., consistent engagement in planned cycling training units per week). This criterion was used to ensure that participants had an established and ongoing training routine relevant to the study context. We have clarified this in the referenced section.

Table 1 - Female participants in the current study are noticeably older than the other cohorts from the previous study. While all are considered "young" (<40 y.o.), if one were to take a matched pair of females from each cohort (previous vs current) in terms of relative FTP, then the years of experience and exposure to training are likely a strong influence on EIH responses, not "fitness level" as it is described in this study. Just a speculative thought.

The reviewer raises an interesting point. However, since we have not acquired any information on the years of training and volume, we are unable to answer this question in the current framework. Future research should investigate the effect of training years (which potentially strongly correlates with fitness level) on exercise-induced pain modulation.

L313-315 - For future reference, there are some nice papers on how to administer the RPE scales with clear instructions to be certain that perceived effort is being measured appropriately (Halperin & Emanuel 2020; Halperin & Visgotsky 2025; Malleron et al. 2023). I always have a habit of signposting to these studies with regard to RPE because it can often be misrepresented in many different areas of research where RPE scales are used.

We thank the reviewer for this suggestion and for highlighting these relevant papers. In the present study, participants were extensively familiarised with the rating of perceived exertion scale, and the instructions closely followed the original definition and recommendations provided by Borg (1998). The scale was presented in accordance with its intended anchor points to promote accurate reporting of perceived exertion. We have now added a citation (Borg, 1998) in the Methods section to clarify this procedure.

(8) Code Availability

Thank you for including open source code in your submission. I am very appreciative of researchers who champion open research principles.

We appreciate the reviewer’s comment and support of open research practices. We have updated the code containing the updated analyses and datasets.

**Reviewer #2 (Recommendations for the authors):**

(1) Core interpretation may not be fully supported by the data

We thank the reviewer for this comment. To investigate the role of fitness level and sex in exercise-induced pain modulation, we have now pooled the samples from the previous and current study and conducted frequentist (linear mixed effects models) analyses. Specifically, we tested for the effect of exercise intensity between the subgroups, as well as (consistent with those conducted in the previous study Nold et al., 2026) testing the effect of fitness level and sex across the pooled data. This allowed us to directly test the effects of fitness level and sex on a common scale rather than infer these effects from separate cohorts. Furthermore, we conducted Bayesian analyses to quantify evidence both for and against the presence of exercise-induced pain modulation and its moderation by fitness, sex, and study sample, utilising the Bayes factors comparing nested models of increasing complexity. In addition, we examined the posterior distributions of the regression coefficients (β) and subgroup-specific exercise effects to estimate the magnitude and uncertainty of these effects. The Bayesian analyses provided some evidence that fitness level moderated exercise-induced pain modulation, but sex did not. Importantly, these findings were consistent with the overall pattern of results obtained in the frequentist analyses. All corresponding statistics in the main manuscript and Supplementary Materials have been updated accordingly. To improve clarity, the pooled analyses are now presented in dedicated Result subsections and accompanied by new figures (Fig. 4 and Fig. 5).

(1.1) Lack of baseline condition

We thank the reviewer for this important comment. We agree that, in the absence of a no-exercise control condition, our design does not allow us to determine whether either exercise condition produced hypoalgesia or hyperalgesia relative to no exercise. Rather, the observed differences reflect the relative effects of high- versus low-intensity exercise on pain ratings.

Our study was not designed to assess the overall effect of exercise compared with rest, but rather to investigate whether exercise intensity influences exercise-induced pain modulation. Specifically, we hypothesised that high-intensity exercise would produce greater pain modulation than low-intensity exercise. This approach was motivated by previous work demonstrating intensity-dependent differences in post-exercise pain responses (Jones et al., 2019; Vaegter et al., 2014).

In response to the reviewer's comment, we have revised the manuscript to clarify this distinction and have replaced several instances of "exercise-induced hypoalgesia" with the more neutral term "exercise-induced pain modulation" where appropriate. We also explicitly acknowledge in the Limitations that the lack of a no-exercise condition precludes conclusions regarding absolute hypoalgesic/hyperalgesic effects of either exercise condition.

(1.2) Lack of internal replication across conditions

We thank the reviewer for this comment. We agree that the observed effect was most pronounced for heat pain at the highest stimulus intensity and was not consistently observed across all stimulus intensities or pain modalities. Importantly, however, this pattern mirrors the findings of our previous study, in which exercise intensity effects were likewise evident for heat pain but not pressure pain. We therefore interpret the current findings as a replication of the modality-specific pattern observed previously rather than an isolated positive result.

To further address concerns regarding robustness and sample size, we have now pooled the data from the previous and current studies and conducted additional frequentist and Bayesian analyses examining the moderating roles of fitness level and sex on exercise-induced pain modulation. These analyses yielded converging evidence that fitness level contributes to variability in exercise-induced pain modulation, thereby strengthening confidence in the overall findings.

With respect to pressure pain, previous meta-analyses have reported comparable or even larger exercise effects for pressure pain than heat pain (Naugle et al., 2012). However, most studies included in these analyses employed handheld pressure algometry, whereas our study used cuff pressure stimulation. Consequently, the pressure pain measures used here may not be directly comparable to those reported in the broader literature.

(1.3) Deviations from the original study and selective use of data

We agree with the reviewer that we have adapted the rating scale to reflect a real-time pain rating. To provide a comprehensive account of participants’ pain experience, we have included analyses corresponding to the visualisations on how online ratings evolve over the course of the pain administered in Supplemental Figure S7 – S8. Specifically, we conducted LMER models with exercise intensity × time on heat and pressure pain ratings, separately (Supplemental Fig. S7). The results yielded a significant interaction of exercise intensity × time on heat (*β* = -0.13, *SE* = 0. 06, *t*(7577) = 2.12, *P* = 0.03) but not pressure pain ratings (*β* = -0.07, *SE* = 0.10, *t*(7956) = -0.71, *P* = 0.48). Furthermore, we conducted LMER models with exercise intensity × time × stimulus intensity on heat and pressure pain ratings (Supplemental Fig. S8), suggesting no significant interaction on pressure pain ratings (*β* = -0.0002, *SE* = 0.004, *t*(2353) = -0.06, *P* = 0.95) but a significant interaction on heat pain ratings (*β* = -0.01, *SE* = 0.003, *t*(2201) = -3.24, *P* = 0.001).

As the reviewer correctly notes, to facilitate comparison with the previous study, we conducted our primary analyses using peak pain ratings. We acknowledge that this approach does not fully capture the richness of the continuous data but allows for comparability.

(1.4) Over-reliance on null results regarding sex differences

We thank the reviewer for this important point regarding the interpretation of null effects, particularly in relation to sex differences. We agree that the absence of a statistically significant difference does not, in itself, provide evidence for equivalence between groups.

To address this issue, we have pooled the samples and supplemented our frequentist analyses with Bayesian analyses. Bayes factors were computed to quantify the relative evidence for the null versus alternative hypotheses, providing a continuous measure of support that complements frequentist inference, particularly in cases of non-significant effects where evidence for the absence of an effect is of interest. Furthermore, posterior distributions of the regression coefficient of interest (in this case, the moderating role of sex on exercise-induced pain modulation) provided no evidence for a moderating role of sex on exercise-induced pain modulation. This approach allows for a more nuanced interpretation of the data, especially concerning null effects. We have adapted the Methods, Results, and Discussion accordingly.

(2) Limited sample and lack of diversity

We thank the reviewer for this comment. The study was intentionally designed to include only female participants with significantly higher fitness levels compared to the females from the previous study, as our previous study included females who were slightly less fit than males, making it difficult to separate the effects of sex and fitness. We, therefore, aimed to examine exercise-induced pain modulation in a more homogeneous sample. However, much of the EIH literature has been conducted in highly trained male participants (Janal et al., 1984; Saanijoki et al., 2022; Scheef et al., 2012). As such, studies in women of higher fitness are important to improve our understanding of potential sex differences in exercise-induced pain modulation. While the present findings should be interpreted within the context of the sample studied, when analysed and considered alongside our previous work, they contribute to a broader understanding of exercise-induced pain modulation across sexes and fitness levels.

(3) Methodological choices limit the interpretability of the data

(3.1) The use of a non-standard VAS scale (0-150 with a fixed pain threshold at 50) is unconventional and may influence how participants report pain, while limiting comparability with related literature.

We thank the reviewer for their comment. Since we introduced real-time pain ratings in the current study, while the pain is ongoing, we aimed to introduce a scale that allowed us to pinpoint the period when a painful stimulus reaches painfulness (exceeds 50 on the scale). To allow for comparability between the studies, we have calculated the peak pain rating provided during the real-time pain ratings, as this corresponds to the instruction participants received in the previous study to rate the most painful point of the previous stimulation. Previous research (Koyama et al., 2004) has shown that, in heat pain, real-time ratings account for R^2^ = 0.89 of the variability of post-stimulus rating, where much of the variability for the post-stimulus intensity pain rating is accounted for by the peak response during the online ratings (adj. R^2^ = 0.3 – 0.4), suggesting good comparability of both pain measures. Furthermore, as we have now pooled the samples and conducted analyses, we rescaled the maximum pain ratings so that ratings below 50 (below the pain threshold) were set to 0, corresponding to VAS 0 in the previous study, resulting in pain ratings ranging between 0 and 100.

(3.2) Participants explicitly reported expecting exercise to reduce pain, introducing a potential confound that is not presently addressed.

We thank the reviewer for this comment. Indeed, participants reported expecting lower whole-body pain in response to acute aerobic exercise, but no changes in joint or muscle pain. In the context of exercise, previous research (Colloca et al., 2018) showed that exercise-unrelated expectations and exercise itself induce comparable hypoalgesic effects, though they might work through distinct mechanisms. Studies directly manipulating exercise-related expectations showed that education about EIH, compared with general education about exercise and pain, could induce hypoalgesia following aerobic exercise (Jones et al., 2017). Interestingly, another study (Vaegter et al., 2020) investigated the interplay of exercise-related expectations (through verbal instructions) and resistance exercise on pain perception and showed that only explicit negative instructions induced hyperalgesia, whereas positive or neutral exercise-related instructions did not change the magnitude of EIH.

Since participants in the current study reported highly specific positive expectations only for whole-body pain, but not for joint or muscle pain, expectations are unlikely to fully explain our findings. Consistent with this, in our previous study using the same prospective expectation questionnaire, we found no significant expectation effects for any pain domain (Nold et al., 2025), although we acknowledge that the samples differed, especially with respect to fitness levels.

Nevertheless, the current study was not designed to disentangle the effects of exercise and expectations, as expectation ratings were measured prospectively but not manipulated. Therefore, we cannot determine to what extent positive expectations of exercise have contributed to the observed effects, and we have now acknowledged this as a limitation in the Discussion.

(3.3) A more comprehensive use of the full time series of pain ratings would provide a stronger and more transparent basis for interpretation of the present findings.

We thank the reviewer for this suggestion. As noted in our response to Comment 1.3, we have included analyses and visualisations of the full time series of online pain ratings in the Supplementary Materials (Fig. S7–S8), providing a more comprehensive understanding of pain dynamics over time.

Figures are very low in quality - artefacts, small text, insufficient labelling.

We thank the reviewer for highlighting this and, indeed, rendering the PDF reduced the quality of the images. For the reviewed version, we have been specifically attentive to image quality by increasing font sizes and adding labels.

There was not enough detail in the methods section for the number of cycling blocks, and how low and high intensity were distributed throughout the session.

We thank the reviewer for their comment. Participants completed four cycling blocks in total, two HI and two LI exercise blocks, presented in a pseudo-randomised order in the session. To improve clarity, we have added the relevant details to the Methods section.
